# Cerebello-Thalamo-Cortical Network Dynamics in the Harmaline Rodent Model of Essential Tremor

**DOI:** 10.3389/fnsys.2022.899446

**Published:** 2022-07-28

**Authors:** Kathryn Woodward, Richard Apps, Marc Goodfellow, Nadia L. Cerminara

**Affiliations:** ^1^School of Physiology, Pharmacology and Neuroscience, University of Bristol, Bristol, United Kingdom; ^2^Department of Engineering, Mathematics and Physical Sciences, University of Exeter, Exeter, United Kingdom; ^3^Living Systems Institute, University of Exeter, Exeter, United Kingdom

**Keywords:** essential tremor, harmaline, cerebellum, thalamus, motor cortex, LFP

## Abstract

Essential Tremor (ET) is a common movement disorder, characterised by a posture or movement-related tremor of the upper limbs. Abnormalities within cerebellar circuits are thought to underlie the pathogenesis of ET, resulting in aberrant synchronous oscillatory activity within the thalamo-cortical network leading to tremors. Harmaline produces pathological oscillations within the cerebellum, and a tremor that phenotypically resembles ET. However, the neural network dynamics in cerebellar-thalamo-cortical circuits in harmaline-induced tremor remains unclear, including the way circuit interactions may be influenced by behavioural state. Here, we examined the effect of harmaline on cerebello-thalamo-cortical oscillations during rest and movement. EEG recordings from the sensorimotor cortex and local field potentials (LFP) from thalamic and medial cerebellar nuclei were simultaneously recorded in awake behaving rats, alongside measures of tremor using EMG and accelerometery. Analyses compared neural oscillations before and after systemic administration of harmaline (10 mg/kg, I.P), and coherence across periods when rats were resting vs. moving. During movement, harmaline increased the 9–15 Hz behavioural tremor amplitude and increased thalamic LFP coherence with tremor. Medial cerebellar nuclei and cerebellar vermis LFP coherence with tremor however remained unchanged from rest. These findings suggest harmaline-induced cerebellar oscillations are independent of behavioural state and associated changes in tremor amplitude. By contrast, thalamic oscillations are dependent on behavioural state and related changes in tremor amplitude. This study provides new insights into the role of cerebello-thalamo-cortical network interactions in tremor, whereby neural oscillations in thalamocortical, but not cerebellar circuits can be influenced by movement and/or behavioural tremor amplitude in the harmaline model.

## Introduction

Essential tremor (ET) is a pathological tremor that affects an estimated ~1% of the population, or 4.6% of those aged 65 years and above (Louis and Ferreira, 2010). ET is characterised as an action tremor—a tremor typically produced by voluntary contraction of muscles and present during sustained posture or voluntary movement (Thenganatt and Jankovic, 2016; Bhatia et al., 2018).

ET tremor frequency in humans is usually around 6–12 Hz and the tremor typically affects the arms and hands but can also affect the head and voice. The pathophysiology underlying ET is unclear, although it is increasingly recognised as a heterogenous disease or syndrome, where there may be several underlying aetiologies producing the clinical phenotype of ET. Converging research suggests that a common feature is abnormalities in activity within the cerebellum which are propagated through the cerebello-thalamo-cortical network. A cardinal symptom of cerebellar dysfunction is intention tremor; a tremor that worsens with goal-directed movement, resulting from disturbances to cerebellar output pathways (Holmes, 1939; Fahn, 1984) and intention tremor has been associated with severe or advanced cases of ET (Deuschl et al., 2000; Louis et al., 2009; Sternberg et al., 2013). Similarly, ataxia—another cardinal sign of cerebellar dysfunction—can be present in ET (Singer et al., 1994; Stolze et al., 2001; Duval et al., 2006; Arkadir and Louis, 2013). Patients with ET have also shown impaired performance on cerebellar-dependent behaviours, such as eye-blink conditioning (Kronenbuerger et al., 2007, 2008), and visuo-motor adaptation (Hanajima et al., 2016). Neuropathological changes have also been observed in the cerebellum of ET patients, including Purkinje cell loss, changes in Purkinje cell morphology and connectivity (e.g., Kuo et al., 2011; 2017a; Yu et al., 2012; Babij et al., 2013; Louis et al., 2014) and redistribution of climbing fibre synapses on the Purkinje cell dendritic arbour, with a greater number of climbing fibre synapses found on distal dendritic spines (Lin et al., 2014; Kuo et al., 2017b; Lee D. et al., 2018; Pan et al., 2020). Clinical studies describing cerebellar abnormalities have, therefore, been closely associated with ET.

A major outflow path of the cerebellum is its projection to the sensorimotor cortex *via* the ventrolateral thalamus (VL), including a subregion termed the ventral intermediate nucleus (VIM, Lierse, 1993). Hua et al. (1998) identified cells within VIM that fire periodically at rates that correlate with tremors. Furthermore, deep brain stimulation of VIM can provide symptomatic relief to ET (Vaillancourt et al., 2003). And thalamic stimulation at a near-to-tremor frequency can entrain the frequency of the tremor, as well as amplify or suppress tremor amplitude, depending on the phase of tremor oscillation when the stimulation is applied (Cagnan et al., 2013). Taken together these findings suggest the thalamus plays an important role in propagating tremor oscillations through the cerebello-thalamo-cortical pathway.

Harmaline is a pharmacological model of tremor, that involves the administration of the β-carboline alkaloid harmaline, which is a reversible inhibitor of monoamine oxidase-A (Hoon et al., 1997). Harmaline-induced tremor is characterised as an action tremor, akin to ET, with the frequency of tremor varying slightly across species (10–16 Hz in mice, 8–12 Hz in rats, 7–12 Hz in monkeys, and 10–16 Hz in pigs; Lamarre et al., 1975; Martin et al., 2005; Lee J. et al., 2018). Harmaline tremor shares similar underlying neural pathways as ET (Handforth, 2012). For example, electrical stimulation within the thalamus in harmaline-treated mice and rats significantly reduces the amplitude of harmaline-induced tremor, akin to the effects of deep brain stimulation observed in ET patients (Bekar et al., 2008; Lee and Chang, 2019). Harmaline induces a temporary increase in the frequency of rhythmic activity in the brainstem inferior olivary (IO) complex, predominantly within the caudal medial accessory olive and caudal dorsal accessory olive (De Montigny and Lamarre, 1973; Lamarre et al., 1975). This in turn, increases the regularity and synchrony of climbing fibre evoked complex spikes in the cerebellar cortex, particularly within the vermis and paravermis, with a complete suppression of simple spikes (De Montigny and Lamarre, 1973; Bernard et al., 1984).

It has previously been hypothesised that tremor-onset with action in ET is due to disrupted cerebellar output (Buijink et al., 2015). The cerebellar nuclei transmit integrated sensorimotor signals to the wider motor network (e.g., Giuffrida et al., 1981; Armstrong and Edgley, 1984; Rowland and Jaeger, 2005; Becker and Person, 2019). Bilateral rhythmic optogenetic stimulation of the interpositus nucleus has been shown to induce a tremor in mice at the same frequency of the stimulation (Brown et al., 2020). This underscores the importance of increased cerebellar nuclear rhythmicity in generating and propagating tremorgenic rhythms. Neuropathological changes in ET may disrupt normal movement-related oscillations in the cerebellum, which in turn may disrupt cerebellar output from the cerebellar nuclei during movement (Pellerin and Lamarre, 1997; Hartmann and Bower, 1998; Courtemanche et al., 2002; Dugué et al., 2009; Baumel et al., 2021). Research to date, however, has not examined the impact of harmaline on cerebellar projections to ascending thalamo-cortical pathways, nor whether movement modulates activity in these pathways. Our aim was to examine harmaline’s effect on the cerebello-thalamo-cortical network in the awake behaving rat by recording local field potential (LFP) oscillations at the tremor frequency across the network and using coherence analysis to examine how these neural network interactions are modulated by movement. Cross-correlation analysis was carried out to examine the functional connectivity of oscillations between nodes of the tremor network. We observed that harmaline-induced tremor can be characterised as an action tremor associated with coherent tremor frequency oscillations across the cerebello-thalamo-cortical network, suggesting propagation of tremor oscillations across the network. Furthermore, harmaline induced prominent pathological oscillations in the cerebellum, and cerebellar coherence with tremor were observed when the animals were resting/immobile and moving. In contrast, thalamic coherence with tremor was modulated by movement. These findings provide evidence that harmaline-induced tremor involves comparable electrophysiological correlates to those reported in ET. Additionally, these findings suggest that the neural oscillations in the cerebellum and thalamus have different roles in modulating tremor, as thalamus but not cerebellar oscillations are influenced by movement and/or behavioural tremor amplitude in the harmaline model.

## Materials and Methods

### Animals

All procedures were performed in accordance with the United Kingdom Animals (Scientific Procedures) Act 1986 and the University of Bristol Animal Welfare and Ethical Review Body. Experiments were performed on 14 adult male Lister Hooded rats (300–600 g). All animals were housed in groups under normal environmental conditions (~20°C and 45%–65% humidity), maintained on a 12/12 h light/dark reverse lighting cycle and provided with food and water ad libitum. Rats were handled daily for at least 1 week prior to surgery. Following surgery, the animals were housed separately, and their health was monitored closely with observational assessments and monitoring of weight.

### Surgery

Rats were anaesthetised with a combination of ketamine (50%) and medetomidine (30%) in saline (20%) delivered via i.p. injection at a dose of 1 ml/kg, and then placed in a stereotaxic frame and secured with atraumatic ear bars coated with local anaesthetic lidocaine (10% Xylocaine^®^). Occasionally an additional dose of 0.1 ml of the ketamine/medetomidine/saline solution was given to maintain surgical levels of anaesthesia, as evidenced by pedal and eye blink reflexes. Core body temperature was maintained at 36–37°C through a rectal thermometer and heat mat.

Previous research has shown the firing rate and rhythmic activity of cells within the medial cerebellar nucleus show a stronger response to harmaline than cells within the interpositus or lateral cerebellar nuclei (Batini et al., 1981; Lorden et al., 1992). Furthermore, the harmaline-induced rhythmic firing of IO neurons occurs mainly in the caudal medial accessory olive and caudal dorsal accessory olive, which provide climbing fibre projections to the vermal A and B zones which then project to the medial cerebellar nuclei and lateral vestibular nucleus (De Montigny and Lamarre, 1973; Llinás and Volkind, 1973; Batini et al., 1981). Therefore, the medial cerebellar nucleus was targeted in these experiments. Rats were implanted with microdrives targeting the medial cerebellar nuclei (1 mm lateral and 11.3 mm posterior from bregma, and 4.1 mm ventral from the surface of the cerebellum) and the ventral anterior and ventral lateral (VA/VL) complex of the thalamus (1.8 mm lateral, 2.28 mm posterior from bregma, and 5.1 mm ventral from the surface of the cerebral cortex), also known as the “motor thalamus” (Nakamura et al., 2014) with two-to-four tetrodes per brain site (impedance 80–400 kΩ at 1 kHz). Two EEG screws were implanted over either side of the motor cortex (3 mm lateral and 2 mm anterior from bregma), and two EEG screws were implanted over either side of the sensory cortex (3 mm lateral and 0.5 mm anterior to bregma).

For 7 out of 14 rats, one of the cerebellar tetrodes was cut ~2 mm shorter to simultaneously target the cerebellar cortex. All tetrodes were coated with fluorescent marker DiI (3% in absolute ethanol) before implantation. Pairs of flexible stainless-steel wires (Cooner wire, USA) were implanted and sutured into the neck EMG and either the triceps brachii or biceps femoris muscles to record EMG. A reference screw pre-soldered to the insulated silver wire was fixed into the right parietal skull plate, and a support screw was fixed into the left parietal skull plate. A ground screw soldered to an insulated silver wire was fixed over the right occipital skull plate. Following the completion of surgery, 1 ml dose of analgesic carprofen (5% in saline) was given subcutaneously followed by 0.1 ml of atipamezole (20% in saline) given *via* i.p. injection, to reverse the effects of the medetomidine.

### Neurophysiological Recordings

After recovery from surgery, differential recordings were made using a tethered CerePlex μ Headstage and a Cereplex acquisition system (Blackrock Microsystems). Raw data were continuously recorded at a sampling rate of 30 kHz and all data were analysed off-line (see the section on “Data Processing”). Low pass filtered EEG and EMG data (<500 Hz) were sampled at 2 kHz. The Blackrock CerePlex μ Headstage also enabled 3-axis accelerometer recordings, which were low pass filtered (<500 Hz) and sampled at 2 kHz. Data were collected before (i.e., pre-harmaline) and after administration of harmaline hydrochloride (10 mg/kg, i.p.). Neural and EMG signals were sampled while rats were quietly at rest or moved freely around their home cage. Baseline data were collected across 2–5 days. After harmaline administration, data were continuously recorded until any visible deficits in motor function (e.g., tremor, ataxia) were completely recovered.

### Histology

Upon completion of the experiment, animals were deeply anaesthetised with Euthatal (1 ml, i.p.) and an electrolytic lesion (3 μA for 30 s) made at the tetrode recording site with the greatest signal-to-noise ratio (SNR). Rats were then transcardially perfused with 0.9% saline, followed by 0.1 M phosphate buffer (PB) that contained 4% paraformaldehyde. Neural tissue was post-fixed in 4% paraformaldehyde for 24–72 h, and then transferred into 30% sucrose solution for 3–4 days before cutting and mounting sections. Cerebellar tissue was cut sagittally at a thickness of either 40 μm (*n* = 10 rats) or 100 μm (*n* = 6 rats). The thalamus was sectioned either in the coronal (*n* = 5 rats; 100 μm section thickness) or sagittal plane (*n* = 10 rats; 40 μm section thickness). Additional verification of tetrode placement within the cerebellum and thalamus was obtained by identifying histological tissue tracks of tetrodes marked with DiI. These were visualised with a fluorescent Axioskop 2 Plus microscope (Zeiss) fitted with a CoolLED pE-100 excitation system and images acquired using AxioVision software.

### Data Processing

Processing of raw data was performed offline using MATLAB (2018) version 9.4.0.813654 (R2018a) Natick, Massachusetts: The MathWorks Inc. Bipolar referencing configurations were used for all EMG, EEG, and tetrode LFP recordings. Where multiple tetrodes were inserted into a single brain region, the tetrode with the greatest SNR of multiunit activity was selected for group analysis. Data were band-pass filtered between 1 and 49 Hz, and then down-sampled to 1 kHz.

A principal component analysis was performed on tri-accelerometer data to combine measurements in three directions into one principal component or axis to capture the axis with maximal variance for subsequent spectral analysis.

To examine how neural network interactions are modulated by movement, electrophysiological data were divided into epochs where the rats were quietly resting vs. moving. This was achieved by applying a global movement-threshold value to a measure of total acceleration, which was the absolute magnitude of acceleration in any direction and was taken as a proxy of overall movement, to distinguish between any periods of resting vs. moving. Total acceleration, *A*, was calculated as:


(1) $$A=\sqrt{x^{2}+y^{2}+z^{2}}$$

Here, *x*, *y*, and *z* represent the three axes of the accelerometer. Total acceleration was then smoothed using a moving average filter with a window size of 100 samples. A global movement threshold of 1 m/s^2^ was then applied to the measure of total acceleration to distinguish periods of resting/immobility from movement (Pasquet et al., 2016; Meyer et al., 2018; Guitchounts et al., 2020). This threshold was verified against video recordings and performed well at identifying time points when rats begin to move around the cage or move to adjust their resting position. Electrophysiological data were then categorised into “resting” or “moving”, whereby rats were classified as either resting or moving if total acceleration, *A*, was below or above the threshold for the entire two-second non-overlapping epoch duration, respectively. Classified epochs were visually verified against the video recordings.

A trade-off between frequency resolution and the number of epochs was introduced when choosing epoch length. A shorter epoch size increased the number of total epochs, whereas a longer epoch size increased the low-frequency resolution of recorded signals but decreased the number of epochs in total. A 2-s-long epoch was chosen as it captured eight cycles of 4 Hz, which is the lowest tremor frequency recorded in patients, as well as providing a good number of epochs for analysis (average = 642 ± 330 epochs per condition per rat). Neural data were collated across baseline and harmaline conditions and collated data were z-score normalised to allow comparison of spectral amplitude across conditions and rats. Epochs containing data points larger than or equal to four standard deviations (SD) of the mean were rejected from further analysis to remove large amplitude artefacts (West et al., 2018).

### Spectral Analysis

For analysis of EMG, accelerometer, and LFP oscillations at the tremor frequency, fast Fourier transforms (FFT) were applied to each 2-s epoch (2,000 data points per epoch). Accelerometer amplitude ratio was defined as the signal amplitude within the tremor frequency range (9–15 Hz) divided by the total amplitude in the low-frequency range (0–15 Hz), calculated for each epoch as follows:


(2) $$Amplitude\;ratio=\frac{\sum Sxx\;(9-15Hz\;bins)}{\sum Sxx\;(0-15Hz\;bins)}$$

Here *Sxx* represents the absolute magnitude obtained from the FFT. Similar methods have been previously employed to examine changes in the distribution of power at tremor-specific frequencies (Iseri et al., 2011). Signal frequency coherence was computed by examining the magnitude-squared coherence (*Cxy*) for each epoch using the “mscohere” function in MATLAB:


(3) $$Cxy=\frac{\left|Pxy\;(f)\right|}{Pxx\;(f)\;Pyy\;(f)}$$

Here *Cxy* represents coherence, *Pxy* represents the cross-spectrum of the two signals and *Pxx* and *Pyy* represent the separate amplitude spectrum of the two signals.

In order to account for levels of coherence solely due to chance, we used a surrogate corrected version of the coherence. We focused on coherence in the tremor frequency band by averaging coherence values in the interval 9–15 Hz. Next, for each epoch, 99 surrogate datasets were generated using the iterative amplitude adjusted fast Fourier transform algorithm (IAAFFT) using MATLAB (Venema et al., 2016). Coherence was computed on each of the 99 surrogate epochs, and the statistical significance of coherence was examined by comparing mean coherence at the tremor frequency (9–15 Hz) for each recorded epoch against the mean coherence at 9–15 Hz for the 99 surrogate epochs. Finally, surrogate-corrected mean coherence values, C, were calculated as Rummel et al. (2010):


(4) $$C=\frac{P-P\;(surr)}{1-P\;(surr)}S$$

Here *P* represents the mean coherence coefficient across the tremor frequency coherence bins (9–15 Hz) of the real data, and *P* (surr) represents the mean coherence at the tremor frequency across 99 surrogate epochs. If *P* is greater than *P* (surr), then the null hypothesis that the two signals are independent can be rejected, and S takes a value of 1. Else, the null cannot be rejected, S takes the value of 0.

### Cross-Correlations

Time-lagged relationships between LFP recorded from each brain region, as well as with EMG, were calculated using cross-correlation analysis. Data were first band-pass filtered at the tremor frequency range (9–15 Hz) before dividing into epochs as described above. Epochs recorded during harmaline conditions when the rat was categorised as “moving” were selected for further analysis. Cross-correlations were calculated using the “xcorr” function in MATLAB. Time-lags showing maximum correlation coefficients were pooled across epochs, and the normalised probability of maximum cross-correlation at binned time-lags was calculated (±100 ms lags with a 2 ms bin width). Peak probability was calculated as the summed probability at the highest bin and the two adjacent probability bins (i.e., the sum at the peak). Peak probabilities that surpassed a threshold value of 0.1 were included within summary statistics examining time-lagged relationships between tremor signals recorded across the network.

### Changes in Coherence Across the Neural Network

To examine changes in coherence across the recorded neural network, statistical analyses compared the area under the mean coherence curve at the tremor frequency (9–15 Hz) between the recorded and surrogate datasets using one-tailed paired *t*-tests. Holm-Bonferroni adjusted p-values were applied to control for multiple coherence comparisons per condition.

### Multilevel Regression Models

Multilevel regression models were fit using MLwiN (v3.05, Centre for Multilevel Modelling, University of Bristol, UK), to investigate the impact of harmaline on LFP amplitude at the tremor frequency and the impact of movement on LFP coherence at the tremor frequency in the harmaline treated rat. Models were specified with the amplitude ratio or surrogate-corrected mean coherence at 9–15 Hz as the response variable, with responses for each epoch (level 1) nested under each rat (level 2). A null model (no explanatory variables) was compared to a random intercepts model which specified a dichotomous explanatory variable for harmaline (harmaline vs. baseline) or movement (movement vs. resting). The random intercepts model was then compared to a random slopes model, which allows the relationship (i.e., slope) between the explanatory and response variables to vary across rats. The inclusion of this level 2 variation can provide a more accurate estimate of the standard error when random-slope variation is present (Bell et al., 2019).

Models are presented in equations 5–7 below, where *y_ij_* is the response variable for rat *j* at sampled epoch *i*. The null model estimates the grand mean of the *y*, represented by *β_0_*, and the variability in the grand mean across rats, *u_0j_*, and sampled epochs *e_ij_*. Model 2 and 3 additionally includes a regression coefficient β_1_ for the dummy coded explanatory variable, *x*, {0, 1}. The regression coefficient for the intercept *β_0ij_* represents the reference group, which is either the control condition when harmaline is included as the explanatory variable, *x*, or resting when movement is included as the explanatory variable, *x*. If the random intercepts models provided a significantly better fit than the null, the random intercepts model (model 2) was then statistically compared to the random slopes model (model 3).


(5) $$\begin{array}{ccc} \text{Model }1:\text{ Null} & \text{Level }1: & y_{ij}=\beta_{0ij}\\ & \text{Level }2: & \beta_{0ij}=\beta_{0}+u_{0j}+e_{ij} \end{array}$$

(6) $$\begin{array}{ccc} \text{Model 2: Random intercepts} & \text{Level }1: & y_{ij}=\beta_{0ij}+\beta_{1}x_{ij}\\ & \text{Level }2: & \beta_{0ij}=\beta_{0}+u_{0j}+e_{ij} \end{array}$$

(7) $$\begin{array}{ccc} \text{Model 3: Random slopes} & \text{Level }1: & y_{ij}=\beta_{0ij}+\beta_{1j}x_{ij}\\ & \text{Level }2: & \beta_{0ij}=\beta_{0}+u_{0j}+e_{ij}\\ & & \beta_{1j}=\beta_{1}+u_{1j} \end{array}$$

If diagnostic checks revealed a non-normal distribution of the residuals in the model, a generalised linear model with an approximate Poisson distribution of the response data was applied and the model was re-run (Leyland and Groenewegen, 2020). Wald chi-squared tests were applied to statistically examine the significance of the effects, testing the null hypothesis that the coefficient for the explanatory variable equals the coefficient for the baseline variable. This statistically compares the standardised regression coefficients for harmaline vs. control or for moving vs. resting.

## Results

### Harmaline Induces a Tremor at 9–15 Hz in Awake Rats

**Figure 1:**
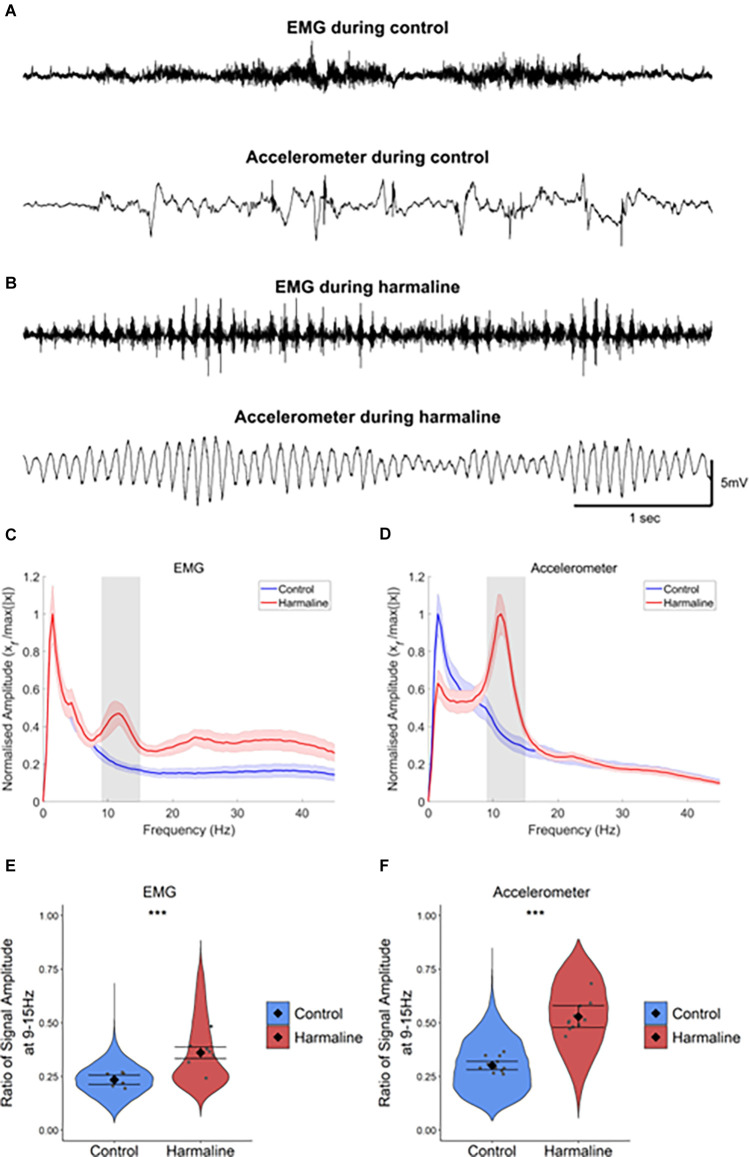
Harmaline induces a 9–15 Hz tremor. **(A,B)** Representative example EMG and accelerometer recording from a rat during **(A)** control and **(B)** harmaline. The upper trace is EMG, and the lower trace is accelerometer. **(C,D)** Mean (±SE) amplitude spectrum for **(C)** EMG (*n* = 7 rats) and **(D)** accelerometer (*n* = 12 rats) during control and harmaline. The solid line represents mean amplitude, and the coloured shaded areas represent SE. The grey area represents the tremor frequency (9–15 Hz). **(E,F)** Ratio of amplitude at the tremor peak for **(E)** EMG and **(F)** accelerometer, where the violin plots show the distribution of this ratio across all epochs (pooled across rats). Individual grey data points represent the mean per rat. Fixed effects parameter estimates (±CI) representing predicted mean estimates are shown by ♦ and corresponding error bars. ^***^ indicates *p* < 0.001.

A whole-body tremor in response to systemic harmaline administration was observed within 5–15 min of intraperitoneal injection of harmaline and lasted for up to 3 h. The tremor could be readily identified in the raw EMG and accelerometer traces (Figures 1A,B). Harmaline induced a tremor at 9–15 Hz with a peak at 11 Hz, as shown in the EMG and accelerometer amplitude spectrum (shaded grey region; Figures 1C,D), where the peak tremor frequency remained relatively stable over time (Supplementary Figure 1). A Wald chi-square test showed the estimated coefficients for EMG and accelerometer amplitude ratio at 9–15 Hz were significantly greater for harmaline (EMG: mean = 0.36, 95% CI [0.33 0.39], Accelerometer: mean = 0.53, 95% CI [0.48 0.58]) than pre-harmaline conditions (EMG: mean = 0.24, 95% CI [0.21 0.26], χ^2^_(1)_ = 4.385, *p* = 0.036, *n* = 7 rats, Accelerometer: mean = 0.30, 95% CI [0.28 0.32], χ^2^_(1)_ = 75.633, *p* < 0.001, *n* = 12 rats; Figures 1E,F). These observations are in agreement with previous studies on harmaline-induced tremor in awake rodents (Pan et al., 2018, 2020).

### Harmaline Induces a Change in Neural Rhythms in Cerebellar Circuits

**Figure 2:**
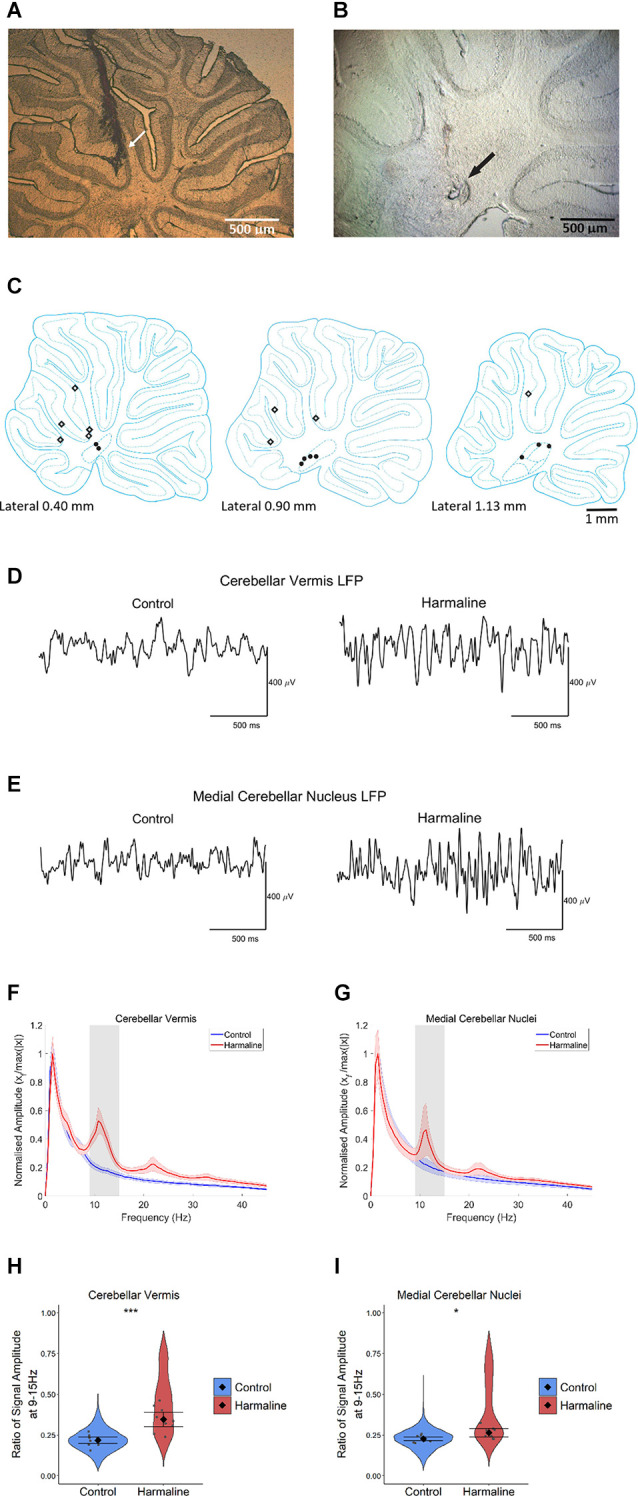
Harmalineincreases oscillatory activity in the cerebellum at the tremor frequency. **(A,B)** Examples of sagittal cerebellar sections with tetrode tracts in the **(A)** cerebellar vermis and **(B)** medial cerebellar nuclei from two different rats. Arrows indicate the approximate positions of the tetrode tips. **(C)** Summary diagram illustrating approximate electrode positions on sagittal sections of the cerebellum (adapted from Paxinos and Watson, 2005). Black dots represent approximate electrode positioning per rat in the medial cerebellar nuclei, and diamond symbols represent approximate electrode positions per rat in the cerebellar cortex. **(D,E)** Representative LFP traces from **(D)** cerebellar vermis and **(E)** medial cerebellar nuclei during pre-harmaline control and harmaline. **(F,G)** Mean (±SE) amplitude spectrum for **(F)** cerebellar vermis (*n* = 9) and **(G)** medial cerebellar nuclei (*n* = 9) during control and harmaline. The solid line represents mean amplitude, and the coloured shaded areas represent SE. The grey area represents the tremor frequency. **(H,I)** Ratio of amplitude at the tremor peak for **(H)** cerebellar vermis (*n* = 9) and **(I)** medial cerebellar nuclei (*n* = 8), where the violin plots show the distribution of this ratio across all epochs (pooled across rats). Individual grey points represent the mean per rat. Fixed effects parameter estimates (±CI) representing predicted mean estimates are shown by ♦ and corresponding error bars. ^***^ indicates *p* < 0.001, and ^*^ indicates *p* < 0.05 **(E,F)**.

Post-mortem histology of the recording site location confirmed that from a total of 14 animals, five animals had cerebellar tetrode recording sites located in the medial cerebellar nucleus, five animals had tetrode recording sites located within the vermal cerebellar cortex and four animals had tetrode recording sites located in both the medial cerebellar nucleus and the vermal cerebellar cortex (Figures 2A–C). The LFP recorded from the cerebellar cortex and cerebellar nuclei across rats during pre-harmaline and harmaline conditions are shown in Figures 2D,E. An increase in rhythmic activity in the tremor frequency range (9–15 Hz; peak 11 Hz, shaded grey region) was seen in both the cerebellar cortex and medial cerebellar nucleus during harmaline compared to the pre-harmaline condition (Figures 2F,G). Smaller amplitude harmonic oscillations are also present at twice the tremor frequency (~23 Hz). Quantitatively, harmaline significantly increased the LFP amplitude ratio at 9–15 Hz in the cerebellar cortex and in the medial cerebellar nuclei (Figures 2H,I; cerebellar cortex: mean = 0.35, 95% CI [0.30 0.39], medial cerebellar nuclei: mean = 0.27, 95% CI [0.24 0.29]) compared to the control condition (cerebellar cortex: mean = 0.22, SE = 0.01, χ^2^_(1)_ = 32.14, *p* < 0.01, *n* = 9 rats, medial cerebellar nuclei: mean = 0.23, 95% CI [0.22 0.24], χ^2^_(1)_ = 8.32, *p* = 0.004, *n* = 8). One rat was excluded from the medial cerebellar nuclei analysis as the mean LFP amplitude for harmaline was greater than two standard deviations of the group mean. Taken together these results suggest that harmaline induces tremor frequency (9–15 Hz) oscillations in the vermal cortex and medial cerebellar nuclei.

### Harmaline Was Not Found to Significantly Increase the Amplitude of Tremor Rhythms in Thalamocortical Circuits

**Figure 3:**
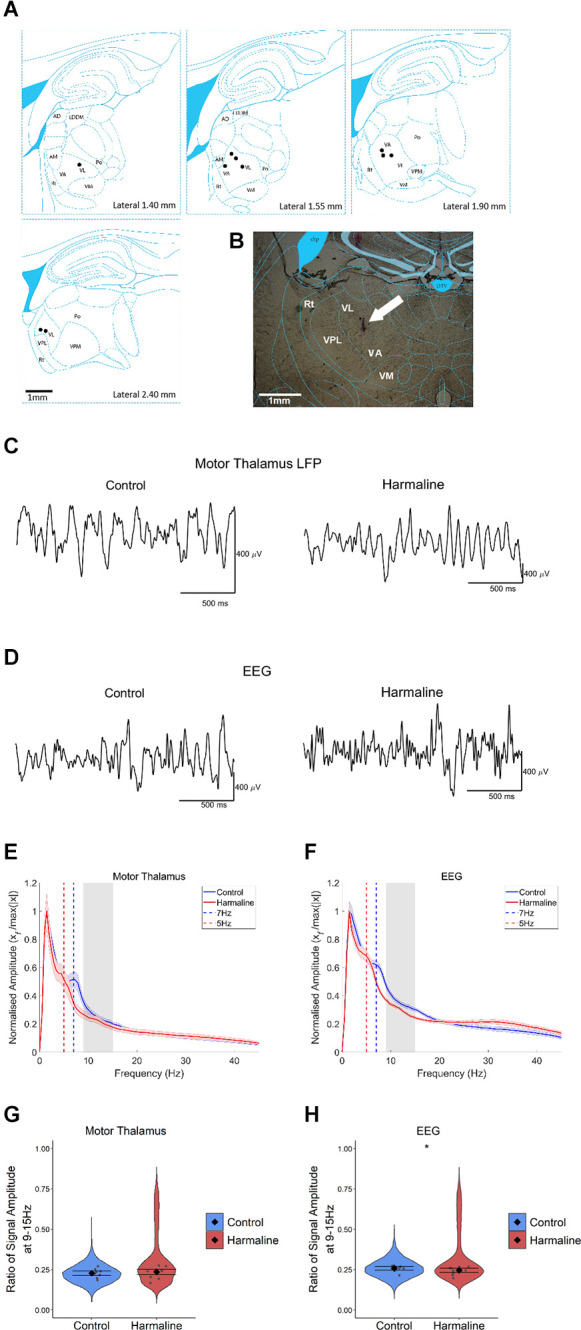
The amplitude of oscillations at the tremor frequency in the thalamocortical circuits. **(A)** Summary diagram illustrating approximate electrode positions on sagittal sections of the thalamus (adapted from Paxinos and Watson, 2005), where each black dot represents the approximate electrode positioning per rat and the VA/VL complex of the thalamus (i.e., motor thalamus). **(B)** Example coronal section from one rat with electrode approximately located in the VA/VL complex of the thalamus. The arrow indicates the approximate position of the tetrode tip. **(C,D)** Representative LFP traces from **(C)** motor thalamus and **(E)** EEG during pre-harmaline control and harmaline. **(E,F)** Mean (±SE) amplitude spectrum for **(E)** motor thalamus (*n* = 9 rats) and **(F)** EEG (*n* = 9 rats) during control and harmaline. The solid line represents mean amplitude, and the coloured shaded areas represent SE. The grey area represents the tremor frequency. **(G,H)** Ratio of amplitude at the tremor peak for **(G)** motor thalamus and **(H)** EEG, where the violin plots show the distribution of this ratio across all epochs (pooled across rats). Individual grey points represent the mean per rat. Fixed effects parameter estimates (±CI) representing predicted mean estimates are shown by ♦ and corresponding error bars. ^*^ indicates *p* < 0.05.

To examine the impact of harmaline on neural activity in thalamocortical circuits, the amplitude spectrum of LFP recorded from the motor region of the thalamus and subdural EEG (Figures 3C,D) recorded over the sensorimotor cortex were assessed. In a total of 10 animals, histological identification of tetrode tracks (Figures 3A,B) indicated that recordings were located within the VL/VA complex of the thalamus, also known as the “motor thalamus”, and we use this term to refer to the VL/VA thalamus complex. Prior to harmaline, a small peak in oscillatory activity at around 7 Hz was observed in both the motor thalamus and the EEG (vertical blue dotted line in Figures 3E,F). However, during harmaline, this 7 Hz oscillation was replaced by a smaller amplitude oscillation at 5 Hz (vertical red dotted line in Figures 3E,F). No distinct peaks in oscillatory activity were detected at the tremor frequency range (9–15 Hz, grey shaded regions in Figures 3E,F).

A Wald test revealed no significant difference in motor thalamus LFP amplitude ratio at 9–15 Hz for harmaline (mean = 0.24, 95% CI [0.22 0.25]) vs. control (mean = 0.23, 95% CI [0.21 0.24], χ^2^_(1)_ = 0.84, *p* = 0.359, *n* = 9; Figure 3G). However, there was a small but significant decrease in EEG amplitude ratio at 9–15 Hz during harmaline (mean = 0.25, 95% CI [0.23 0.26]) compared to the pre-harmaline condition (mean = 0.26, 95% CI [0.25 0.27]), (χ^2^_(1)_ = 3.85, *p* = 0.05, *n* = 9; Figure 3H). These findings illustrate a change in the rhythmicity of thalamocortical activity during harmaline vs. control conditions, where there is a shift from 7 Hz to 5 Hz.

### Harmaline-Induced Tremor Amplitude Is Modulated by Movement

**Figure 4:**
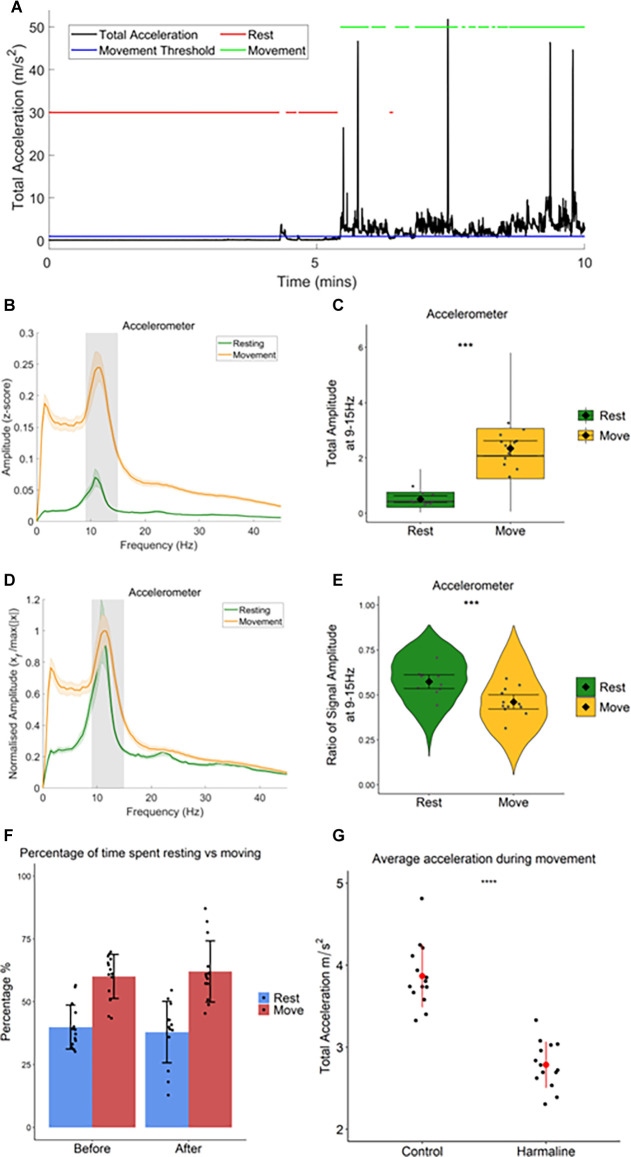
Totalamplitude of harmaline-induced tremor increases withmovement.** (A)** Movement threshold (1 ms^−2^; bluehorizontal line) applied to total acceleration data (black) todistinguish periods of rest and movement. Red lines demonstrate timepoints categorised as “rest”, and green lines demonstrate timepoints categorised as “movement”. **(B)** Total (±SE) and**(D)** relative (±SE) amplitude accelerometer spectrum (*n* = 12 rats) during rest and movement. The solid line represents the mean, and the coloured shaded areas represent SE. The grey area represents the tremor frequency. **(C)** Total accelerometer amplitude and **(E)** ratio of accelerometer amplitude at the tremor peak, across all epochs (pooled across rats). Individual grey points represent the mean per rat. Fixed effects parameter estimates (±CI) representing predicted mean estimates are shown by ♦ and corresponding error bars. ^***^ indicates *p* < 0.001. **(F)** Percentage of time spent resting and moving during baseline (before) and harmaline conditions. Each dot represents one rat. Error bars represent ±SD. **(G)** Average total acceleration during movement before (control) and after harmaline treatment. Each dot represents one rat. Error bars represent ± SD. ^***^ indicates *p* < 0.001.

As harmaline tremor is reported to be an action tremor that resembles ET (Pan et al., 2018, 2020), it is of interest to examine how activity and interactions within the tremor-related neural network are modulated by movement. To examine whether harmaline-induced tremor changes in severity during movement, accelerometer activity was compared during periods of movement vs. resting, which was defined by total acceleration falling above or below a threshold of 1 m/s^2^, respectively, for the entire epoch duration (Figure 4A). In both behavioural states, the accelerometer spectrum has a distinct peak at the tremor frequency range (9–15 Hz, grey banded section of each panel (Figure 4B). A Wald test showed that total accelerometer amplitude at the tremor frequency was significantly greater during movement (mean = 2.35, 95% CI [2.07 2.62]) than during rest (mean = 0.51, 95% CI [0.39 0.63], χ^2^_(1)_ = 166.42, *p* < 0.001, *n* = 12; Figure 4C).

Figure 4B also illustrates that across the entire spectrum (<45 Hz) accelerometer amplitude was greater during movement than during rest. To ensure that the difference in tremor frequency amplitude between resting and moving was not related to the generalised increase in accelerometer amplitude during movement, the amplitude ratio at the tremor frequency was also examined (see “Methods” Section) to compare the relative amplitude of oscillations at 9–15 Hz (Figure 4D). This revealed a significant increase in the ratio of accelerometer tremor frequency amplitude for rest (mean = 0.57, 95% CI [0.054 0.61]) compared to movement (mean = 0.46, 95% CI [0.42 0.50], χ^2^_(1)_ = 31.66, *p* < 0.001, *n* = 12; Figure 4E), indicating that the increase in total accelerometer amplitude during movement was not specific to the tremor frequency range.

In summary, these results, therefore, suggest that harmaline-induced tremor is present when rats are at rest and also during movement, but the amplitude of rhythmic activity across the frequency spectrum studied (0–45 Hz) increases significantly with movement. This corresponds with the visual inspection of tremor, where a low amplitude tremor was visible during resting, but the tremor became much more pronounced when the rat moved around the cage.

In addition to harmaline producing a significant action tremor in rats, general ataxia was also observed 5–15 min following the intraperitoneal injection and could last for up to 3 h. This included a loss of coordination, an unsteady gait, and an increased spreading of the paws and foot slips during walking. There was also an absence of rearing and an increase in occurrences where the rat was lying down or leaning on the side of the cage. However, when the percentage of time rats spent actively moving vs. being quietly at rest was compared there was no statistically significant difference between pre-harmaline (median = 62.0%) and harmaline (median = 59.6%, *z* = −0.408, *p* = 0.683, *r* = −0.109, *n* = 14 rats). This suggests that harmaline has little or no effect on overall activity levels in rats (Figure 4F). However, rats’ movements were, significantly slower (on average by 28%) during harmaline [mean = 2.79 m/s^2^, SD = 0.28] in comparison to control conditions [mean = 3.87 m/s^2^, SD = 0.38, t_(13)_ = 9.883, *p* < 0.001, *n* = 14 rats; Figure 4G].

### Harmaline-Induced Changes in Coherence During Movement and Rest

To examine the degree to which neural oscillations across the cerebello-thalamo-cortical network correlate with harmaline-induced behavioural tremor, coherence between the neural activity recorded from each of the three brain regions under investigation and tremor activity measured *via* the accelerometer were assessed during rest and movement following harmaline administration.

**Figure 5:**
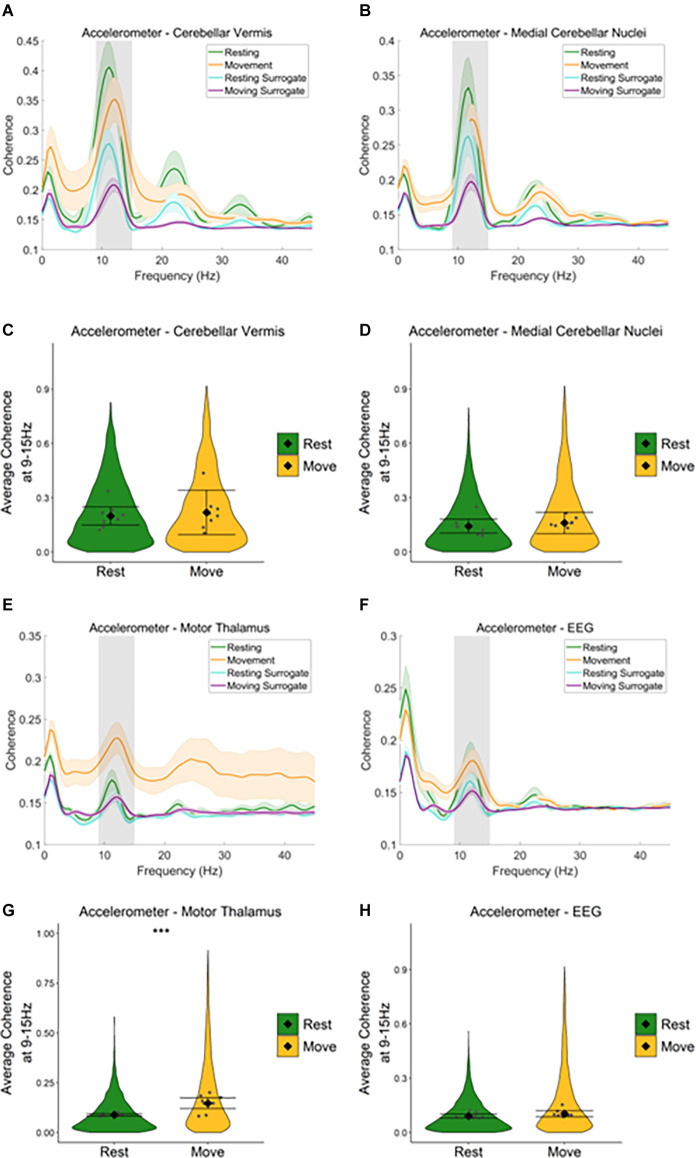
Neural oscillations across the cerebello-thalamo-cortical network correlate with harmaline-induced behavioural tremor. **(A,B)** Mean (±SE) **(A)** cerebellarcortex-kinematic (*n* = 8 rats), **(B)** cerebellar nuclei-kinematic (*n* = 7 rats) during resting and movement for real and surrogate datasets. The solid line represents meancoherence, and the coloured shaded areas represent SE. The grey area represents the tremor frequency. **(C,D)** Average surrogate-corrected **(C)** cerebellarcortex-kinematic, **(D)** cerebellar nuclei-kinematic coherence at the tremor peak across all epochs with significant coherence (pooled across rats). Individual greypoints represent the mean per rat. Fixed effects parameter estimates (±CI) representing predicted mean estimates are shown by ♦ and corresponding error bars. ^***^indicates *p* < 0.001 **(E)** thalamic-kinematic ((textitn = 8 rats), and **(F)** cortico-kinematic (textitn = 6 rats) coherence during resting and movement for real and surrogate datasets. The solid line represents mean coherence, and the coloured shaded areas represent SE. The grey area represents the tremor frequency. **(G,H)** Averagesurrogate-corrected **(G)** thalamic-kinematic and (H) cortico-kinematic coherence at the tremor peak across all epochs with significant coherence (pooled acrossrats). Individual grey points represent the mean per rat. Fixed effects parameter estimates (±CI) representing predicted mean estimates are shown by ♦ andcorresponding error bars. ^***^ indicates *p* < 0.001.

During rest and movement, a peak in coherence between the accelerometer and the cerebellar vermis LFP (Figure 5A), and the accelerometer and medial cerebellar nuclear LFP (Figure 5B) was evident at the tremor frequency (9–15 Hz, grey shaded area). A harmonic peak in coherence at double the tremor frequency (~23 Hz) is also evident, which is likely due to a non-sinusoidal neural oscillation at the tremor rhythm. Surrogate analysis revealed that 76.6% of epochs showed significant cerebellar vermis-kinematic coherence at 9–15 Hz in comparison to the surrogate dataset (*n* = 8,142 out of 10,632 epochs, which included 76.2% of epochs classified as “resting”, and 77.0% of epochs classified as “moving”). The analysis also revealed that 65.0% of epochs also showed significant medial cerebellar nuclear-kinematic coherence at 9–15 Hz (*n* = 6,478 out of 9,963, this included 64.7% of epochs classified as “resting”, and 65.0% of epochs classified as “moving”). Overall, this shows average coherence at 9–15 Hz was greater for the recorded data (Figures 5A,B; green and yellow lines) compared to surrogate datasets (Figures 5A,B, blue and purple lines).

Model coefficients for surrogate-corrected mean coherence were compared across resting and movement (Figures 5C,D). This revealed no difference in surrogate-corrected mean coherence at the tremor frequency across resting and movement, for either cerebellar vermis-kinematic coherence (Rest: mean = 0.20, 95% CI [0.15 0.25], Movement: mean = 0.22, 95% CI [0.10 0.34], χ^2^_(1)_ = 0.28, *p* = 0.599, *n* = 8) nor medial cerebellar-nuclei-kinematic coherence (Rest: log mean = −1.93, 95% CI [−1.19 −1.68], mean = 0.14, 95% CI [0.10, 0.18] Movement: log mean = −1.84, 95% CI [−1.91, −1.58], mean = 0.16, 95% CI [0.10, 0.22], χ^2^_(1)_ = 0.16, *p* = 0.684, *n* = 7).

Together, the surrogate analysis, therefore suggests that tremor-frequency oscillations in the cerebellar vermis and medial cerebellar nuclei are significantly correlated with kinematic measures of tremor in harmaline-treated rats for the majority of epochs (76.2% for the cerebellar cortex, 65.0% for the cerebellar nuclei). Furthermore, epochs showing statistically significant coherence are distributed equally across resting and movement, and the strength of coherence is not modulated by movement.

Equivalent analysis of the motor thalamus and EEG data also revealed a peak in coherence between the accelerometer and motor thalamic LFP (Figure 5E), and the accelerometer and EEG (Figure 5F) at the tremor frequency (9–15 Hz, grey shaded area) during rest and movement. However, the peak coherence was lower than that found for the cerebellar vermis and medial cerebellar nuclei. Broader peaks in coherence were also observed at double the tremor frequency (~24 Hz). Surrogate analysis revealed that 54.5% of epochs showed significant motor thalamo-kinematic coherence at 9–15 Hz in comparison to the surrogate dataset (*n* = 5,597 out of 10,267 epochs, 49.0% of “resting” epochs, 61.8% of “moving” epochs), where average motor thalamo-kinematic coherence was greater for recorded data compared to surrogate datasets (Figure 5E). Comparison of surrogate-corrected mean coherence coefficients also revealed a significant increase in tremor frequency coherence for movement (log mean = −1.93, 95% CI [−2.25, −1.29], mean = 0.15, 95% CI [0.11, 0.28]) compared to resting (log mean = −2.41, 95% CI [−2.53, −2.30], mean = 0.09, 95% CI [0.08, 0.10], χ^2^_(1)_ = 28.07, *p* < 0.001, *n* = 8; Figure 5G). Surrogate analysis also showed 52.3% of epochs showed significant EEG-kinematic coherence at 9–15 Hz in comparison to the surrogate dataset (*n* = 6,402 out of 12,249 epochs, 50.82% of “resting” epochs, 54.0% of “moving” epochs). However, there was no significant difference in surrogate-corrected mean coherence coefficients at the tremor frequency for movement (log mean = −2.26, 95% CI [−2.29, −1.97], mean = 0.10, 95% CI [0.09, 0.12]) vs. resting epochs (log mean = −2.382, 95% CI [−2.52, −2.24], mean = 0.09, 95% CI [0.08, 0.10]), χ^2^_(1)_ = 2.47, *p* = 0.116, *n* = 9; Figure 5H).

**Figure 6:**
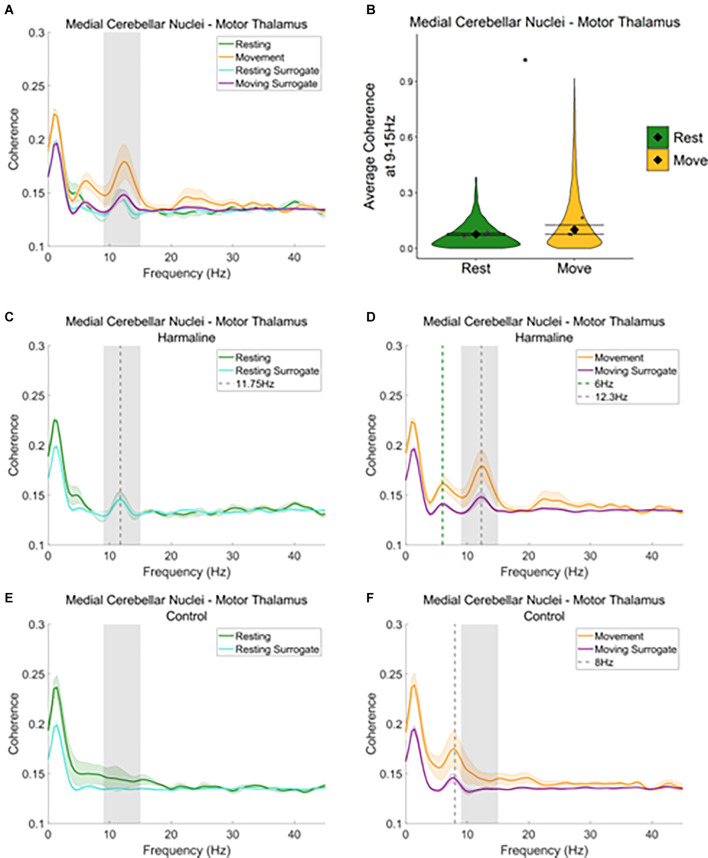
Cerebello-thalamiccoherence in the harmaline model.** (A)** Mean (±SE)**(A)** cerebellar nuclei-thalamic (*n* = 6) coherenceduring resting and movement for real and surrogate datasets. Thesolid line represents mean coherence, and the coloured shaded areasrepresent SE. The grey area represents the tremor frequency.**(B)** Average surrogate-corrected cerebellar nuclei-thalamiccoherence at the tremor peak across all epochs with significantcoherence (pooled across rats). Individual grey points represent themean per rat. Fixed effects parameter estimates (±CI)representing predicted mean estimates are shown by ♦ and corresponding error bars. ^*^ indicates *p* < 0.05. **(C–F)** Mean (±SE) cerebellar nuclei-thalamic (*n* = 6); coherence in harmaline model **(C,D)** and control **(E,F)** during resting **(C,E)** and movement **(D,F)** for real and surrogate datasets. The solid line represents mean coherence, and the coloured shaded areas represent SE. The grey area represents the tremor frequency.

**Figure 7:**
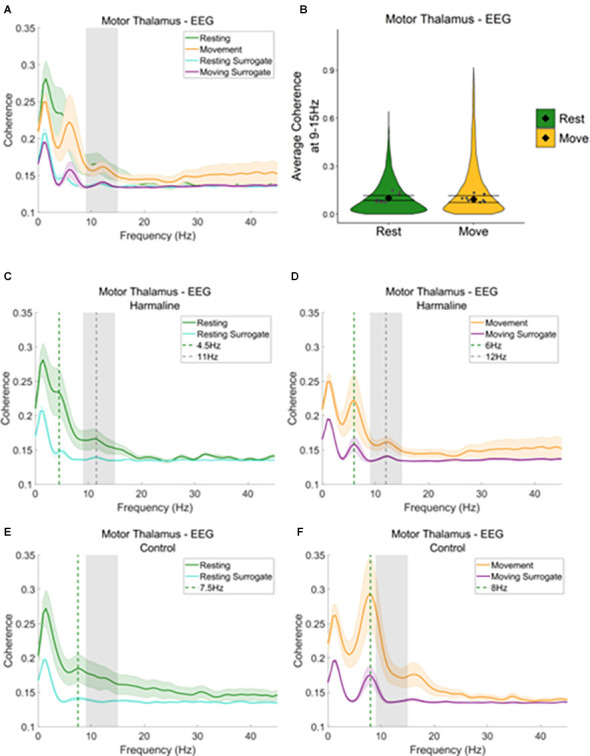
Thalamocorticalcoherence in the harmaline model.** (A)** Mean (±SE) thalamocortical coherence (*n* = 8) during resting and movement for real and surrogate datasets. The solid line represents mean coherence, and the coloured shaded areas represent SE. The grey area represents the tremor frequency. **(B)** Average surrogate-corrected thalamocortical coherence at the tremor peak across all epochs with significant coherence (pooled across rats). Individual grey points represent mean per rat. Fixed effects parameter estimates (±CI) representing predicted mean estimates are shown by ♦ and corresponding error bars. **(C–F)** Mean (±SE) thalamocortical coherence (*n* = 8); coherence in harmaline model **(C,D)** and control **(E,F)** during resting **(C,E)** and movement **(D,F)** for real and surrogate datasets. The solid line represents mean coherence, and the coloured shaded areas represent SE. The grey area represents the tremor frequency.

A similar pattern was seen when examining average coherence values at the tremor frequency for medial cerebellar nuclei-thalamic and motor thalamus-EEG coherence (Figures 6 and Figure 7). A peak in medial cerebellar nucleo-motor thalamic coherence was observed at the tremor frequency (Figure 6A), with coherence at this frequency greater for recorded data compared to surrogate datasets during movement only, and not during rest (Figures 6C,D). A total of 47.3% of epochs showed significant medial cerebellar nucleo-motor thalamic coherence at 9–15 Hz in comparison to the surrogate dataset (*n* = 3,635 out of 7,681 epochs, 44.7% of “resting” epochs, 49.2% of “moving” epochs). There was also a significant increase in surrogate-corrected medial cerebellar nucleo-motor thalamic coherence for movement (log mean = −2.31, 95% CI [−2.36, −1.74], mean = 0.10, 95% CI [0.08, 0.13]) compared to resting (log mean = −2.58, 95% CI [−2.77, −2.39], mean = 0.08, 95% CI [0.07, 0.08], χ^2^_(1)_ = 5.79, *p* = 0.016, *n* = 6; Figure 6B). In addition to the peak in medial cerebellar nucleo-motor thalamic coherence at the tremor frequency, a small peak was also observed at approximately half the tremor frequency during movement (grey and green dotted lines, Figure 6D). During the control condition, a peak in medial cerebellar nucleo-motor thalamic coherence was observed at ~8 Hz, during movement only (green vertical dotted line; Figure 6F) and not during rest (Figure 6E), which may reflect an intrinsic movement-related oscillation.

When examining motor thalamus-EEG coherence, a small peak in coherence was also found at the tremor frequency range (9–15 Hz; Figure 7A, grey dotted line Figures 7C,D), where coherence was greater for recorded data compared to surrogate datasets. A total of 52.6% of epochs showed significant motor thalamus-EEG coherence at 9–15 Hz in comparison to the surrogate dataset (*n* = 5,685 out of 10,802 epochs, 52.1% of “resting” epochs, 53.1% of “moving” epochs). However, there was no significant difference in surrogate-corrected mean coherence coefficients at the tremor frequency for movement (log mean = −2.36, 95% CI [−2.62, −2.26], mean = 0.09, 95% CI [0.07, 0.11]) compared to rest (log mean = −2.28, 95% CI [−2.46, −2.10], mean = 0.10, 95% CI [0.09, 0.12], χ^2^_(1)_ = 0.21, *p* = 0.646, *n* = 8; Figure 7B). In addition to the small 9–15 Hz peak in motor thalamus-EEG coherence, a much larger peak in coherence was evident at 4.5–6 Hz, which may reflect an intrinsic thalamocortical oscillation or a sub-harmonic of the tremor frequency. This peak was more prominent during movement than rest (green dotted line, Figures 7C,D). To examine whether the 4.5–6 Hz oscillation was related to harmaline-induced tremor, or an intrinsic thalamocortical oscillation, thalamocortical coherence during baseline control conditions for both movement and rest was also inspected (Figures 7E,F). During the control condition, a peak in motor thalamus-EEG coherence was observed at ~8 Hz, where coherence at this frequency is greater during movement than rest (green vertical dotted line; Figures 7E,F). This suggests the presence of an intrinsic thalamo-cortical oscillation at ~8 Hz during control conditions, which is modulated by motor activity. During harmaline tremor, the frequency of this oscillation shifts to ~4.5–6 Hz (Figure 7D; green vertical dotted line), which could be an intrinsic thalamocortical oscillation or reflect a sub-harmonic of the tremor frequency.

In sum, these findings illustrate significant motor thalamo-kinematic coherence at the tremor frequency for 54.5% of epochs (Figure 5G) that was significantly modulated by movement despite the absence of a peak in thalamic oscillatory LFP activity at the tremor frequency during harmaline conditions compared to control (Figure 3E). Furthermore, the strength of medial cerebellar nuclear-motor thalamic coherence was significantly modulated by movement (Figure 6B), even though the strength of medial cerebellar nuclear-kinematic coherence at the tremor frequency was not modulated by movement (Figure 5D). Significant sensorimotor EEG-kinematic coherence and motor thalamus-EEG coherence at the tremor frequency was also found, but this was not significantly modulated by movement, with evidence suggesting that harmaline can induce a change in the frequency of intrinsic thalamocortical rhythms, whereby a ~8 Hz rhythm is shifted to 4.5–6 Hz rhythm. Together these findings suggest a role for thalamic involvement in harmaline-tremor rhythms during motor activity.

### Changes in Motor Thalamic LFP During Movement

**Figure 8:**
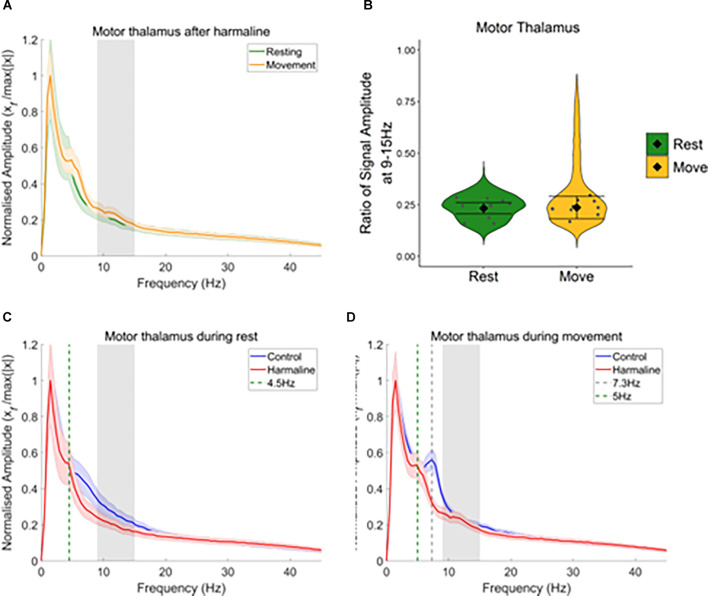
Harmalinechanges the frequency of thalamus rhythms during movement. **(A)** Mean (±SE) amplitude spectrum for the motor thalamus (*n* = 9) during rest and movement during harmaline. The solid line represents mean amplitude, and the coloured shaded areas represent SE. The grey area represents the tremor frequency. **(B)** Ratio of amplitude at the tremor frequency range for the motor thalamus, where the violin plots show the distribution of this ratio across all epochs. Individual grey points represent the mean per rat. Fixed effects parameter estimates (±CI) representing predicted mean estimates are shown by ♦ and corresponding error bars. **(C,D)** Mean (±SE) amplitude spectrum for the motor thalamus (*n* = 9) during **(C)** rest and **(D)** movement during control and harmaline. The solid line represents mean amplitude, and the coloured shaded areas represent SE. The grey area represents the tremor frequency.

As significant motor thalamic-kinematic coherence was observed during harmaline conditions, and as this coherence significantly increased during movement vs. rest, the amplitude spectrum of motor thalamic LFP during harmaline conditions was also compared across rest and movement epochs (Figure 8A). A Wald test revealed no significant difference in motor thalamus LFP amplitude ratio at 9–15 Hz for rest (mean = 0.23, 95% CI [0.21 0.26]) vs. movement (mean = 0.24, 95% CI [0.18 0.29], χ^2^_(1)_ = 2.66, *p* = 0.064, *n* = 9; Figure 8B). The lack of changes in LFP amplitude at the tremor frequency was further explored by comparing LFP amplitude during rest and movement during pre-harmaline control and harmaline conditions (Figures 8C,D). An oscillation at ~7 Hz was found to be present during the pre-harmaline control condition when the rats were moving (Figure 8D, grey dotted line) but not when the rats were resting (Figure 8C, blue line). During harmaline, this ~7 Hz oscillation during movement shifted to a ~5 Hz oscillation (Figure 8D, green dotted line). A smaller peak at approximately 5 Hz could also be seen during rest for harmaline conditions (Figure 8C). This shift in oscillation frequencies may be due to an intrinsic 7–8 Hz movement-related oscillation which is entrained to a sub-harmonic of the tremor frequency during harmaline.

**Table 1:** Surrogate analysis of coherence at the tremor frequency (9–15 Hz).

	**Control**				**Harmaline**			
**Paired Connection**		**During resting**		**During movement**		**During resting**		**During movement**	
		*t*	*p*	*t*	*P*	*t*	*p*	*t*	*p*	*N*
Cb—Acc		2.73	0.015	2.28	<0.028^*^	5.45	<0.001^*^	3.86	0.003^*^	8
CN—Acc		1.29	0.122	3.29	<0.008^*^	3.38	<0.007^*^	7.80	<0.001^*^	7
Thal—Acc		−1.23	0.871	3.50	<0.005^*^	2.71	<0.015^*^	4.41	0.002^*^	8
EEG—Acc		0.83	0.215	7.01	<0.001^*^	3.37	<0.005^*^	4.18	0.002^*^	9
CN—Thal		1.23	0.136	1.84	<0.062^*^	0.53	<0.308^*^	2.31	0.034^*^	6
Thal—EEG		2.20	0.032	3.29	<0.012^*^	3.38	<0.034^*^	3.18	<0.001^*^0‥	8

Paired one-tailed t-tests were applied to statistically examine whether the area under the mean coherence curve at the tremor frequency was greater than the area under the mean coherence curve at the tremor frequency from surrogate datasets. N, number of rats included in analysis; Acc, Accelerometer; Cb, Cerebellar cortex; CN, Medial Cerebellar nuclei; Thal, Thalamus. ^*^Significant following Holm-Bonferroni-adjusted p-values to control for family-wise error rate.

### Changes in Network Coherence

**Figure 9:**
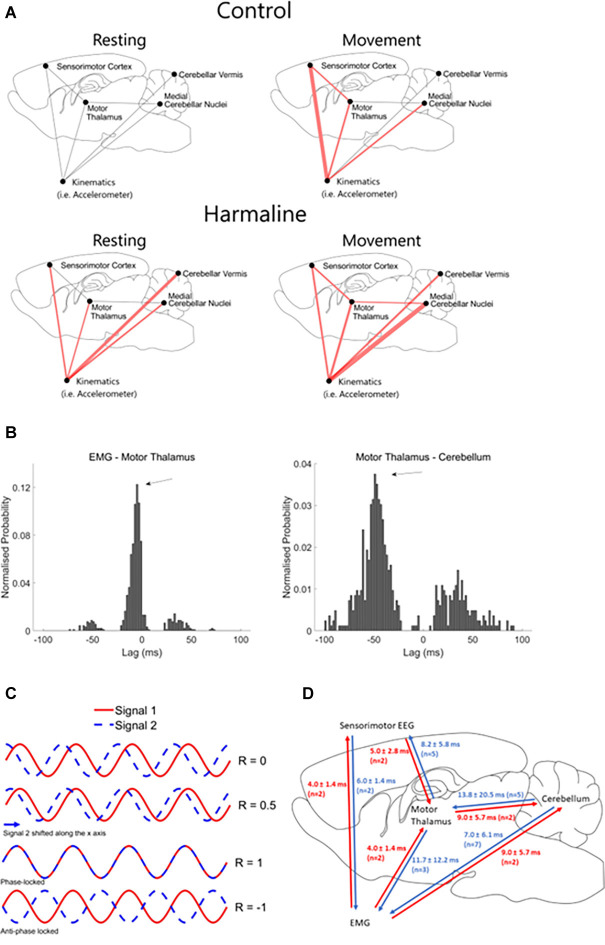
Changes in connectivity across the medial cerebellar-motor thalamus-cortical network. **(A)** Surrogate analysis of coherence at the tremor frequency (9–15 Hz) during control (rest and move) and harmaline (rest and move). Red lines indicate significantly greater coherence at 9–15 Hz for the recorded neural data compared to the mean from 99 surrogate datasets generated using IAAFT, with Holm-Bonferroni adjusted significance levels for multiple comparisons. The width of the red line corresponds with the size of the t-statistic of the one-tailed paired *t*-tests comparing surrogate and original datasets, where the thicker the line the larger the effect-size. **(B)** Example histogram displaying the probability of time-lags (ms) with max cross-correlation across 2-s epochs taken during motor activity after harmaline administration for a single rat. Examples are from two different rats, and negative lags correspond to a backward connection between the name pairs. Arrows indicate time-lags with peak probability of max cross-correlation. **(C)** Schematic of cross-correlation at different time lags for two perfect sine waves at the same frequency. Shifting one signal (e.g., signal 2) relative to the other (e.g., signal 1) influences the correlation coefficient (R), which varies between −1 (negative correlation) and 1 (positive correlation). A perfect positive correlation is found when the signals are perfectly phase-locked (i.e., perfect alignment of the two sine waves). A perfect negative correlation between these two signals is found when signals are perfectly locked in an anti-phase direction (i.e., the peaks of signal 1 aligning with troughs of signal 2, and vice versa). **(D)** Schematic showing mean time delays of tremor frequency oscillations across each recording node in the tremor-network. N corresponds to the number of rats included in the summary statistic.

To examine differences in the mean magnitude of coherence at the tremor frequency at a network level, statistical analyses were applied to compare the mean area under the coherence curve at 9–15 Hz for real and surrogate datasets (Table 1), and the results are summarised graphically in Figure 9A. During control (i.e., no harmaline) conditions, there was no statistically significant coherence in the motor network at the tremor frequency range when the rats were quietly at rest. However, motor activity under control conditions (without tremor) was associated with significant 9–15 Hz coherence between the kinematic measure and: (1) the sensorimotor cortex (EEG); (2) the medial cerebellar nuclei; and (3) the motor thalamus, as well as thalamocortical (motor thalamus-EEG) coherence. Coherence at this frequency range in the absence of tremor suggests the presence of an intrinsic movement-related neural oscillation in the motor network occurs within a similar frequency range as harmaline-induced tremor. This corresponds with data presented in Figure 7F; which identified a thalamocortical oscillation occurring within the theta frequency range (~8 Hz) during movement for control (non-tremor) conditions.

Following administration of harmaline, statistically significant coherence at the tremor frequency (9–15 Hz) was evident at rest between all brain regions and the kinematic measure of tremor. However, no statistically significant tremor-related activity was present within the cerebello-thalamo-cortical pathway at rest. By comparison, during movement, statistically significant tremor-related coherence was found across the entire network. In sum, both motor activity and harmaline-induced tremor are associated with increased coherence at 9–15 Hz, but coherence across the medial cerebellar nuclear-motor thalamo-cortical pathway is dependent on the presence of tremor during active movement (Figure 9A).

To examine the time-lags of oscillations within the tremor network, cross-correlations were performed. Normalised probability histograms of time-lags with max cross-correlation were computed per rat (see “Methods” Section), and the time-lags with peak probability surpassing the probability threshold of 0.1 were extracted. Time lags with the maximum cross-correlation (−1 or 1) represent the time lag with the best fit between the two time-series (Figure 9C). This is estimated by shifting the recorded time-series from one node of the tremor network either forwards or backwards relative to another node in the network, which gives a positive or negative time-lag, respectively. Time-lags of the max-correlation are, therefore, classified as being in a positive or negative direction, which indicates whether one time-series may lead or trail the other, to examine the directionality of oscillations and estimate how long it takes for an oscillation to propagate from one region to another (Figure 9C). Figure 9B displays examples of normalised probability histograms of time-lags for different network connections in two different rats. Importantly, these histograms show clear peaks in the probability of max correlation at certain time-lags (arrows in Figure 9B).

The mean (±SD) time-lags for each connection within the tremor network were examined across rats summarised in Figure 9D). In seven out of nine rats, estimated time-lags of simultaneous cerebellar LFP-EMG recordings suggest tremor oscillations travelled from the cerebellum to the muscle (i.e., positive time-lags suggesting forward connection). Five out of seven rats showed positive time-lags between tremor oscillations recorded simultaneously from the cerebellum and motor thalamus LFP, and five out of seven rats also showed positive time-lags between tremor oscillations recorded from motor thalamus LFP and EEG. This suggests that the direction of tremor oscillations largely follows the direction of information flow within the cerebello-motor thalamo-cortical pathway. However, estimated time-lags of oscillations simultaneously recorded across the thalamus LFP-EMG and EEG-EMG, suggest a more complex combination of efferent and afferent inputs of tremor oscillations, as ~50% of these connections showed a positive time-lag, and ~50% showed a negative time-lag.

## Discussion

Our results demonstrate that harmaline-tremor in rats is associated with significant coherence across the medial cerebellar nuclear-motor thalamo-cortical network at the tremor frequency (9–15 Hz). This corresponds with electrophysiological findings from ET patients, which demonstrated that neural oscillations in the thalamus and EEG correlate with behavioural tremor (Hua et al., 1998; Schnitzler et al., 2009). Harmaline is known to increase oscillatory activation of the olivo-cerebellar pathway. Therefore, these results suggest that increased oscillatory activation of this pathway in rats is associated with similar electrophysiological correlates of tremor in ET. The experiments reported here also showed that movement is associated with significantly increased coherence of motor thalamic neural oscillations with harmaline-induced behavioural tremor. Conversely, medial cerebellar nuclear and vermal cerebellar cortex neural tremor oscillations were not modulated by movement or increased tremor amplitude.

### Harmaline as a Model of ET

Previous research has qualitatively described harmaline-induced tremor as an action tremor (Placantonakis et al., 2004; Handforth, 2012), akin to the action tremor phenotype observed with ET (Bhatia et al., 2018). Our results support this observation, demonstrating that harmaline tremor significantly increases in amplitude during movement compared to rest. However, a significant harmaline-induced behavioural tremor was also present during rest/immobility. This could be attributed to either a resting tremor (i.e., a tremor occurring when muscles are completely relaxed), or to a postural tremor, as the threshold used to distinguish rest vs. movement could not differentiate between rest with and without postural muscle control of the head, body, or limbs. Harmaline would therefore seem to provide a good model of action tremor, although there are phenotypic differences from ET. This includes the tremor frequency (9–15 Hz in the harmaline rodent model, and 4–12 Hz in ET; Bhidayasiri, 2005), and the body parts affected by tremor, as harmaline induces a whole-body tremor whereas ET typically affects only the upper limbs and sometimes the head (Bhatia et al., 2018).

Our results also show that harmaline-induced tremor shares similar electrophysiological correlates to ET. Harmaline was shown to induce significant coherence of tremor frequency oscillations across the cerebello-thalamo-cortical network. Previous studies examining cerebral cortico-muscular coupling in ET patients using simultaneous EEG (or MEG) and EMG recordings, as well as direct thalamic electrophysiological recordings, suggest that ET is a disorder involving hyper-synchronous activity across the cerebello-thalamo-cortical circuit (Schnitzler et al., 2006; Muthuraman et al., 2012; Pedrosa et al., 2014). The results reported here, therefore, highlight that harmaline-induced tremor can also be characterised by increased coupling across the cerebello-thalamo-cortical network at the tremor frequency during movement, which suggests propagation of neural tremor oscillations across this pathway.

Although harmaline-induced tremor and ET show similarities in their phenotype and electrophysiological correlates, differences in the aetiology of tremor remain. Harmaline has been well-documented to produce a tremor by increasing the rhythmicity and frequency of olivo-cerebellar inputs (for a review see Handforth, 2012). However, there is little evidence for the involvement of hyper-oscillatory olivo-cerebellar activity in ET. Although there is some neuroimaging evidence indicating overactivity of portions of the brainstem, possibly reflecting the inferior olive (IO, Hallett and Dubinsky, 1993; Boecker et al., 1996), this has not been replicated in other studies (Wills et al., 1994). A post-mortem analysis also failed to reveal any detectable differences in IO cell packing density or evidence of IO neuronal damage in ET compared to controls (Louis et al., 2013). However, gross anatomical differences may not be apparent given that pathophysiological hyperactivity of the IO is thought to underlie the tremor. On the other hand, there is accumulating evidence of heterogeneous Purkinje cell-related pathologies in ET (see Section “Introduction”), indicating a clear role for cerebellar abnormalities in ET (Louis, 2016). These abnormalities may arise from a complex range of degenerative and compensatory changes in cerebellar circuits that would lead to dysregulation and/or increased oscillatory cerebellar nuclei output. This in turn could alter nucleo-olivary projections, which modulate the synchrony and timing of IO neurons (Lefler et al., 2014). Changes in cerebellar nuclei output could also lead to pathological rhythms transmitted to the cerebello-thalamo-cortical pathway, which is hypothesised to be involved in ET (Hua et al., 1998; Buijink et al., 2015). Therefore, despite the likelihood that ET develops due to a complex range of degenerative and compensatory changes in cerebellar circuits, the findings reported here show that the harmaline model provides good construct validity as it increases rhythmic cerebellar output and generates tremor correlated neural oscillations in the thalamocortical network, as well as generating an action tremor.

Overall, our findings show that harmaline-induced tremor is associated with increased oscillatory medial cerebellar nuclear output and tremor-correlated neural oscillations in the motor thalamo-cortical network. However, it is also important to note that the effect of harmaline on the central and peripheral nervous system are non-specific, and little is known about its influence on the wider nervous system. Harmaline is a mono-amine-oxidase inhibitor for group A amines, and therefore inhibits the breakdown of mono-amine neurotransmitters (e.g., noradrenaline, serotonin), which can affect multiple and distributed neural systems (Chen and Shih, 1997; Herraiz et al., 2010). Harmaline may also have acetylcholinesterase inhibitor effects (Udenfriend et al., 1958); acetylcholine receptors are widespread in the cerebellum, and some motor dysfunctions have been related to cerebellar cholinergic dysfunctions (Zhang et al., 2016). Therefore, in addition to inducing tremor *via* its effects on the olivo-cerebellar circuit, harmaline may be inducing unknown effects on the wider nervous system.

### Thalamic Involvement in Tremor

Our results illustrate that harmaline was associated with significant motor thalamo-kinematic coherence at the tremor frequency, indicating thalamic oscillations correlated with behavioural tremor. This is despite a lack of harmaline-induced changes in motor thalamic LFP oscillations at the tremor frequency. Previous research has shown electrical stimulation of the VA/VL nuclei of the thalamus in harmaline-treated mice and rats significantly reduces the amplitude of harmaline-induced tremor, akin to the effects of deep brain stimulation observed in ET patients (Bekar et al., 2008; Lee and Chang, 2019). Harmaline tremor amplitude is also significantly reduced by intra-thalamic infusion of the GABA receptor agonist muscimol, suggesting inhibition of thalamic activity reduces tremor (Bekar et al., 2008). Furthermore, inactivation of adenosine A1 receptors blocked the therapeutic effect of thalamic stimulation on harmaline-treated mice, suggesting that the release of ATP and activation of adenosine A1 receptors may be key to the depression of excitatory transmission in the thalamus and reduction of tremor in response to thalamic stimulation (Bekar et al., 2008). A selective adenosine A1 receptor agonist has been shown to significantly reduce extracellular levels of glutamate in VA/VL thalamic nuclei in rats, as well as significantly reduce the amplitude of harmaline-induced tremor (Kosmowska et al., 2020). Taken together, this suggests the thalamus may play an important role in modulating the amplitude of harmaline-induced tremor in rats.

Our results also show that motor thalamo-kinematic coherence was significantly modulated by movement or increased tremor amplitude in response to movement. This is consistent with findings in ET patients which showed thalamo-muscular coherence occurred after tremor onset (Pedrosa et al., 2014), and that tremor-related rhythmic activity of neurons in the ventral thalamus was only present during tremor induced by sustained posture and not during resting (Hua and Lenz, 2005). This suggests tremor-related oscillations in the thalamus occur after the onset of action tremor, and that thalamo-muscular coherence may be a consequence of tremor rather than a driver. Functional mapping of the ventral thalamus in ET patients revealed that thalamic neurons which burst in correlation with tremor included neurons that responded to sensory input as well as neurons responding to voluntary movements (Hua and Lenz, 2005). This suggests peripheral sensory inputs may be involved in modulating or amplifying tremor-related activity in the thalamus. Therefore, increased motor thalamo-kinematic coherence during movement compared to resting may be at least partly related to sensory feedback of behavioural tremor.

By comparison, medial cerebellar nuclear-kinematic coherence and vermal cerebellar cortex-kinematic coherence were equally strong during movement and rest. As total tremor amplitude significantly increased during movement, this may suggest an extra-cerebellar source is involved in the modulation of tremor amplitude and thalamic tremor-oscillations with movement. For example, afferent feedback of behavioural tremor may amplify thalamic tremor-oscillations. Mechanoreceptors in the skin, muscle, and joints receive information on touch, vibration and proprioception, and these receptors project *via* the dorsal column medial-lemniscal pathway to the dorsal column nuclei complex (DCN), which comprises the gracile and cuneate nuclei (Loutit et al., 2021). The DCN complex in turn has excitatory projections to the ventral posterior lateral and ventral posterior medial (i.e., somatosensory) nuclei of the thalamus (Kramer et al., 2017; Uemura et al., 2020), as well as projections to the zona incerta, red nucleus, cerebellar cortex, and IO (Boivie, 1971; Robinson et al., 1987; McCurdy et al., 1998; Quy et al., 2011). Therefore, the combination of somatosensory feedback of tremor and direct cerebellar-thalamic projections may contribute to the amplification or spread of tremor oscillations in the thalamus during movement.

### Harmaline Induces a Shift in Thalamocortical Oscillation Frequencies

Our findings also show a shift in neural oscillation frequencies identified within the motor thalamus LFP and EEG amplitude spectra in response to harmaline, where oscillations at ~7 Hz in the motor thalamus LFP and EEG during pre-harmaline control conditions are shifted to oscillations at around ~5 Hz during harmaline tremor. In addition, when examining medial cerebellar nuclear-motor thalamus coherence, and thalamocortical coherence, a peak in coherence was observed at 8 Hz during pre-harmaline control, which shifted to a peak at 4.5–6 Hz during harmaline tremor. This shift in the frequency of thalamocortical neural oscillations in response to harmaline could be due to the entrainment of intrinsic ~7–8 Hz rhythms to a sub-harmonic of the tremor frequency, as 5–6 Hz is approximately half the tremor frequency. This would provide further support for thalamic involvement in harmaline tremor pathways and may account for the lack of changes observed in thalamic-EEG coherence at the tremor frequency, as tremor-frequency-related oscillations may instead manifest at a sub-harmonic of the tremor frequency.

We also observed a ~7–8 Hz oscillations during pre-harmaline control conditions in motor thalamus LFP when rats were moving and not resting. A recent study by Baumel and Cohen (2021) demonstrated that a 7–8 Hz theta oscillation in the cerebellar nuclei was higher in power when the animals were moving compared to rest. Therefore, cerebellar output during movement may shape thalamic oscillations and may account for the intrinsic movement related cerebellar nuclear oscillations that we observed, although the functional relevance of this interaction is unknown.

### Roles of the Cerebellum and Thalamus in Modulating Tremor

As the findings presented in this study show that thalamic but not cerebellar oscillations are associated with tremor amplitude, this may indicate that different parts of the cerebello-thalamo-cortical loop have different roles in modulating tremor. Evidence from brain stimulation studies suggests that the cerebellum may be responsible for maintaining the frequency of tremor in ET. For example, transcranial alternating currents applied over the cerebellum at the same frequency as the patient’s tremor did not affect the amplitude of the tremor but could entrain the tremor frequency, i.e., influence the phase and instantaneous frequency of the tremor (Brittain et al., 2015). Research has also shown that tremor frequency in ET is more tuned to a central frequency compared to PD, where tremor frequency can vary over a broader range (Di Biase et al., 2017). In the harmaline model, the frequency of the tremor is also tightly centred on a narrow-frequency range, and *in vivo* electrophysiological studies have shown that this frequency is paced by olivo-cerebellar rhythms (De Montigny and Lamarre, 1973; Llinás and Volkind, 1973). Taken together, this suggests that abnormalities occurring within olivo-cerebellar circuits can produce very regular pathological rhythmic oscillations that are tightly coupled with tremor frequency but are independent of behavioural state.

Comparatively, studies suggest that thalamic oscillations may be tightly coupled with tremor amplitude. For example, thalamic oscillations in the harmaline model were associated with changes in tremor amplitude with movement, and previous clinical research has shown that tremor amplitude in ET was amplified or suppressed depending on the phase of thalamic stimulation at frequencies close to the tremor frequency (Cagnan et al., 2013). Furthermore, DBS applied to the thalamus is used as a chronic treatment for ET, where high frequency (e.g., 150 Hz) stimulation is applied to disrupt or mask tremor oscillations in the thalamus (Kiss et al., 2002; Karas et al., 2013). Taken together, this may suggest abnormal tremor-related neural oscillations in the cerebellum, and possibly descending pathways from the brainstem that receive cerebellar input (e.g., rubrospinal, vestibulospinal, reticulospinal), may play an important role in the timing or pacing of tremor rhythms. However, changes in the synchronisation of thalamo-cortical oscillations to the behavioural tremor rhythm may be important for modulating the amplitude of behavioural tremor, where increased synchronisation at the tremor frequency exacerbates tremor. Sensory feedback may represent one mechanism driving the synchronisation of thalamic oscillations to the behavioural tremor during movement (Hua and Lenz, 2005; Pedrosa et al., 2014). This is counter to traditional theories of ET neural networks, which suggest that the cerebello-thalamo-cortical pathway plays a key role in peripheral tremor (Manto, 2008; Buijink et al., 2015; Muthuraman et al., 2018). However, the findings reported here demonstrated that coherence between the motor thalamus and tremor was modulated by movement, whereas coherence between the cerebellum (medial cerebellar nuclei and cerebellar vermis) and tremor was not, and suggests that whilst the frequency of the tremor may be governed by the cerebellum, thalamic oscillations relate to the amplitude of the behavioural tremor.

## Data Availability Statement

The raw data supporting the conclusions of this article will be made available by the authors, without undue reservation.

## Ethics Statement

The animal study was reviewed and approved by University of Bristol Animal Welfare and Ethical Review Body.

## Author Contributions

NC, RA, and MG conceived and designed the experiment. KW acquired and analysed the data. KW and NC prepared the first draft of the manuscript and the figures. All authors reviewed the manuscript for intellectual content. All authors contributed to the article and approved the submitted version.

## Funding

This work was supported by the Medical Research Council UK (G1100626) and the BBSRC (BB/P000959/1) to NC and RA. This work was supported in part by grant MR/N0137941/1 for the GW4 BIOMED MRC DTP, awarded to the Universities of Bath, Bristol, Cardiff and Exeter from the Medical Research Council (MRC)/UKRI.

## Conflict of Interest

The authors declare that the research was conducted in the absence of any commercial or financial relationships that could be construed as a potential conflict of interest.

## Publisher’s Note

All claims expressed in this article are solely those of the authors and do not necessarily represent those of their affiliated organizations, or those of the publisher, the editors and the reviewers. Any product that may be evaluated in this article, or claim that may be made by its manufacturer, is not guaranteed or endorsed by the publisher.

## Supplementary Material

Supplementary Figure 1 Changes in tremor amplitude over time. Mean accelerometer amplitude spectrum for one rat at different time points following systemic administration of harmaline (10 mg/kg, I.P).
