# Supplementary figures and images for: Cerebello-Thalamo-Cortical Network Dynamics in the Harmaline Rodent Model of Essential Tremor
Source: Front Syst Neurosci. 2022 Jul 28;16:899446. doi: 10.3389/fnsys.2022.899446 (PMC9365993; doi:10.3389/fnsys.2022.899446)

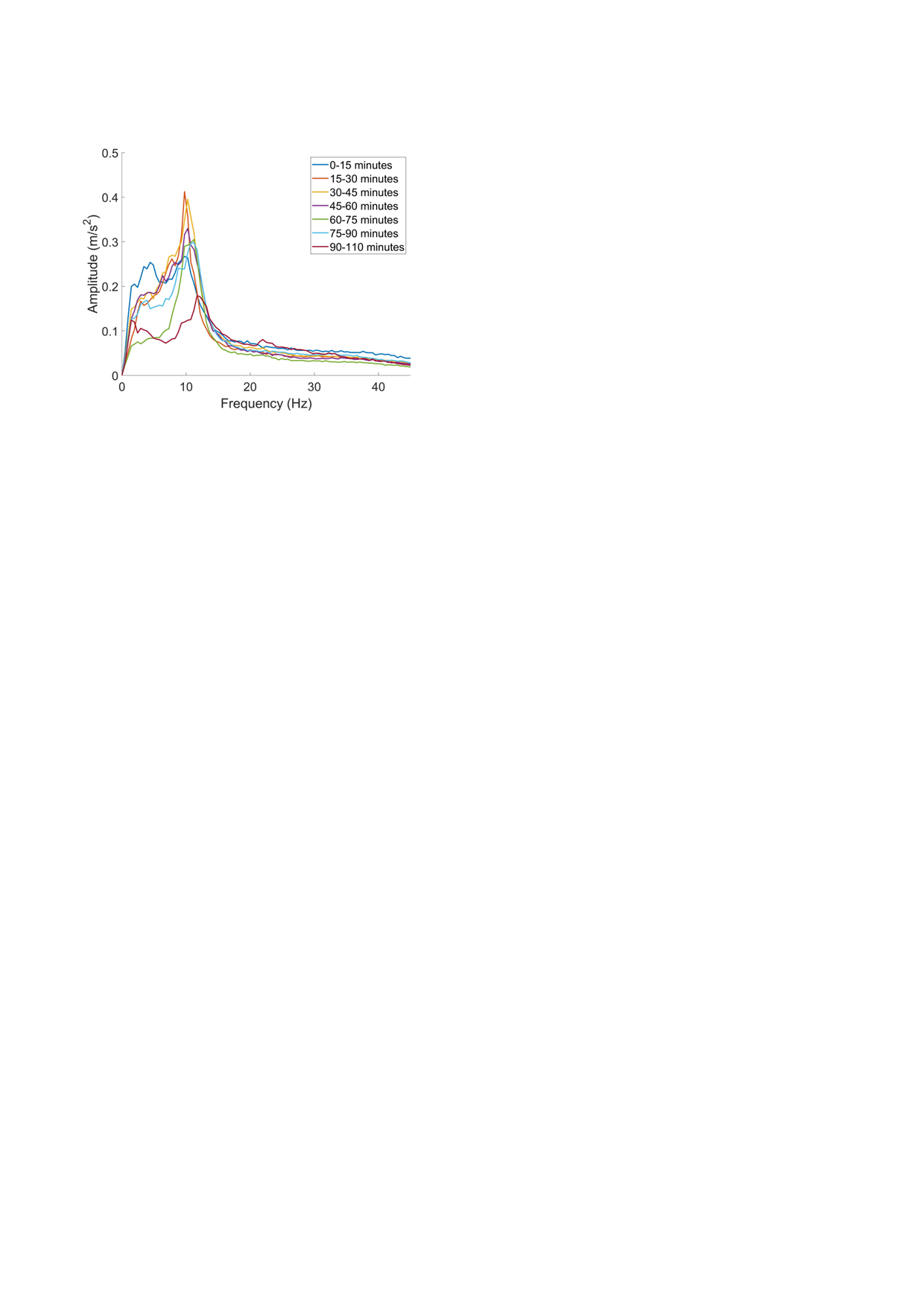

Supplement: Supplementary Figure 1 — Changes in tremor amplitude over time. Mean accelerometer amplitude spectrum for one rat at different time points following systemic administration of harmaline (10 mg/kg, I.P). [file Image_1.TIF]
